# Using High-Pressure Technology to Develop Antioxidant-Rich Extracts from Bravo de Esmolfe Apple Residues

**DOI:** 10.3390/antiox10091469

**Published:** 2021-09-15

**Authors:** Mário Bordalo, Inês J. Seabra, Andreia Bento Silva, Ana Paula Terrasso, Catarina Brito, Margarida Serra, Maria R. Bronze, Catarina M. M. Duarte, Mara E. M. Braga, Hermínio C. de Sousa, Ana Teresa Serra

**Affiliations:** 1 iBET, Instituto de Biologia Experimental e Tecnológica, Apartado 12, 2781-901 Oeiras, Portugal; mjbordalo@gmail.com (M.B.); aterrasso@inovegene.pt (A.P.T.); anabrito@ibet.pt (C.B.); mserra@ibet.pt (M.S.); mbronze@ibet.pt (M.R.B.); 2 Instituto de Tecnologia Química e Biológica António Xavier, Universidade Nova de Lisboa (ITQB NOVA), Av. da República, 2780-157 Oeiras, Portugal; abentosilva@ibet.pt (A.B.S.); cduarte@itqb.unl.pt (C.M.M.D.); 3 CIEPQPF, Department of Chemical Engineering, Faculty of Sciences and Technology (FCTUC), University of Coimbra, Rua Sílvio Lima, Pólo II—Pinhal de Marrocos, 3030-790 Coimbra, Portugal; js218@lehigh.edu (I.J.S.); marabraga@eq.uc.pt (M.E.M.B.); hsousa@eq.uc.pt (H.C.d.S.); 4 Bioengineering Department, Lehigh University, Bethlehem, PA 18015, USA; 5 iMed.ULisboa, Instituto de Investigação do Medicamento, Faculdade de Farmácia da Universidade de Lisboa, 1649-003 Lisboa, Portugal; 6 DCFM, Departamento de Ciências Farmacêuticas e do Medicamento, Faculdade de Farmácia da Universidade de Lisboa, Av. das Forças Armadas, 1649-003 Lisboa, Portugal

**Keywords:** Bravo de Esmolfe, apple, antioxidants, CO_2_ extraction, phenolic compounds, Caco-2 cells, neurospheroids

## Abstract

Bravo de Esmolfe (BE) is a traditional Portuguese apple highly appreciated by consumers due to its peculiar flavor and aroma. This apple contains higher concentration of phenolic compounds than other cultivars and is thus considered a rich source of antioxidants. Its sensorial and functional properties have attracted farmers’ associations to increase BE production. However, a large quantity of apples is wasted due to storage/transportation procedures that impact BE’s quality attributes. In this work, we applied high-pressure extraction methodologies to generate antioxidant-rich fractions from BE residues aiming at adding high value to these agro-food by-products. We performed a first extraction step using supercritical CO_2_, followed by a second extraction step where different CO_2_ + ethanol mixtures (10–100% *v*/*v*) were tested. All experiments were carried out at 25 MPa and 50 °C. Extracts were characterized in terms of global yield, phenolic content and antioxidant activity using chemical (ORAC, HOSC, HORAC) and cell-based assays (CAA). We demonstrated that, although the pressurized 100% ethanol condition promoted the highest recovery of phenolic compounds (509 ± 8 mg GAE/100 g BE residues), the extract obtained with 40% ethanol presented the highest CAA (1.50 ± 0.24 µmol QE/g dw) and ORAC (285 ± 16 µmol TEAC/g dw), as well as HOSC and HORAC values, which correlated with its content of epicatechin and procyanidin B2. Noteworthy, this fraction inhibited free radical production in human neurospheroids derived from NT2 cells, a robust 3D cell model for neuroprotective testing.

## 1. Introduction

Bravo de Esmolfe (BE) is a traditional Portuguese apple variety only produced in a restricted and small inland region called “Beiras” in the centre–northern Portugal. This apple is classified as a “Protected Designation of Origin” product and it is very appreciated by consumers due to its peculiar flavour and aroma [1,2]. In a previous work, we demonstrated that BE apple contains a higher concentration of phenolic compounds and a higher (up to 2-fold) antioxidant capacity than other apple varieties, namely, Golden, Starking, Fuji and Gala Galaxy [3,4,5]. The main phenolic compounds identified in BE were catechin, epicatechin, chlorogenic acid, quercetin glucosides and procyanidins B1 and B2; its antioxidant activity (ORAC value) ranged from 1503 to 2089 µM TEAC/100 g fresh weight (fw) [5]. In particular, the catechin and epicatechin content of the BE variety was found to be 10- and 4-fold higher, respectively, than the above-mentioned apple cultivars [3,5]. Due to its sensorial and functional properties, the cultivation of BE apple has increased and, today, over 6 thousand tons are produced per year. However, storage and transportation procedures impact on the quality attributes of this apple variety; thus, a large quantity of these fruits is wasted every year [1]. Therefore, there is a need to develop strategies to efficiently extract bioactive (e.g., antioxidant) compounds from these agro-food residues with the aim to develop high-added value food ingredients from BE with health-promoting effects.

The recovery of bioactive compounds from agro-food industry wastes should not add more environmental issues when considering the extraction process to be employed. The use of green technologies, together with bio-solvents (e.g., ethanol from biomass) and allied to the exploitation of agro-industrial residues, figures, nowadays, as a matter of paramount importance concerning environmental protection and sustainability [6]. In this field, high-pressure technology, including supercritical fluid extraction (SFE) using CO_2_, has been recognized a promising green process to extract bioactive compounds for food, pharmaceutical and cosmetic applications since it holds several advantages, namely, higher selectivity and shorter extraction times, as well as not using toxic organic solvents [7,8]. CO_2_ is usually the most desirable solvent for extraction as it is generally recognized as safe (GRAS). However, the main drawbacks of CO_2_ are (i) its nonpolar and lipophilic nature and (ii) its inability to extract compounds with high molecular weight, such as flavonoids. Thus, the use of suitable cosolvents has been explored to enhance the solubility of the target compounds and/or to increase the extraction selectivity [9]. Another strategy to overcome this problem is the application of enhanced solvent extraction methodologies (ESE). These techniques usually involve the use of CO_2_, water and/or organic solvents at high temperatures (40–200 °C) and pressures (3.3–20.3 MPa) and have been applied with success by our group and others to the extraction of polar solutes [10], including flavonoids from elderberry pomace [11] and cherry culls [12].

High-pressure technology has already been applied for the extraction of phenolic compounds from apple products. In the study of Adil et al. (2007), the authors optimized the subcritical (CO_2_ + ethanol) extraction of phenolic compounds and antioxidants from apple pomace by varying the pressure, temperature, ethanol concentration and extraction time [13]. The best conditions were achieved for 54.6–57 MPa, 55.7–58.4 °C, 20% (*v*/*v*) ethanol and 40 min, respectively, which allowed the generation of an extract with a phenolic compounds content of 0.47 mg GAE/g sample and a radical scavenging capacity of 3.30 mg DDPH/mg sample. More recently, the SFE at 25 MPa and 50 °C with CO_2_ + ethanol (75:25 mol ratio) was also applied to dried peels of the Golden Delicious variety. In particular, Massias and co-workers (2015) were able to produce extracts with higher phenolic content (up to 50 mg GAE/g sample) and enriched in phloridzin and quercetin derivatives [14].

The present study aims to further explore the use of high-pressure technology to develop antioxidant-rich fractions of apple residues from the BE variety. A fractioned high-pressure extraction was performed at 25 MPa and 50 °C and the methodology employed comprised a first extraction step with supercritical CO_2_, to remove nonpolar and lipophilic substances, followed by a second ESE step, where different mixtures of CO_2_ + ethanol (10–100%, *v*/*v*) were tested as a more polar supercritical extraction phase. We demonstrate, for the first time, that the polar ESE solvent mixture composition impacts on the BE extracts yield, phenolic content, antioxidant activity and neuroprotective effect in human neurospheroids derived from NT2 cells.

## 2. Materials and Methods

### 2.1. Apple Samples

*Bravo de Esmolfe* apple residues were provided by Cooperativa Agrícola de Mangualde. These samples were the fruits that were not suitable to sell due to appearance defects, including small-sized apples. The samples were crushed in a knife mill, then dehydrated in a freeze-drier (Freeze Dryer Modulyo, Edwards, UK) at −40 °C, in the absence of light. After 72 h, the raw material was milled in a grinder (Braun, KSM 2, Kronberg, Germany) and stored at −20 °C until the day of the extraction experiments.

### 2.2. Materials

Carbon dioxide industrial grade (99.5%, Praxair, Madrid, Spain) and pure ethanol (99.5%, Panreac Quimica SA, Barcelona, Spain) were used for the extraction experiments. Other chemicals and solvents employed in phytochemical analysis were o-phosphoric acid (Panreac, Spain), acetonitrile (Fisher, UK), ethanol (Panreac, Spain) and Folin–Ciocalteu’s phenol reagent (Merck KGaA, Germany). Catechin, epicatechin and chlorogenic acid from Merck KGaA (Germany) and procyanidin B2, quercetin-3-glucoside and quercetin-4-glucoside were purchased from Extrasynthese (France).

Chemicals used for antioxidant activity assays were 2′,2′-azobis (2-amidinopropane) dihydrochloride (AAPH), 6-hydroxy-2,5,7,8-tetramethylchroman-2-carboxylic acid (Trolox), caffeic acid (97%) and FeCl_3_, CoF_2_ and hydrogen peroxide from Merck KGaA (Germany) and disodium fluorescein (FL) from TCI Europe (Belgium). Sodium chloride, potassium chloride and potassium phosphate, all from Merck KGaA (Germany), and sodium phosphate dibasic dehydrate (99.5%) from Riedel-de-Haën (Seelze, Germany) were used for the phosphate buffer solution and sodium phosphate buffer preparations (PBS and SPB).

Cell culture media, supplements and reagents used in cell-based assays included RPMI 1640, DMEM, fetal serum bovine (FBS), penicillin-streptomycin (P/S), MTT 3-(4,5-Dimethylthiazol-2-yl)-2,5-Diphenyltetrazolium Bromide (MTT) and Presto blue™, all purchased from Thermo Fisher Scientific (USA). Quercetin (>95%) and 2′,7′-Dichlorofluorescin diacetate (DCFH-DA) were acquired from Merck (Germany).

### 2.3. Extractions

#### 2.3.1. High-Pressure Extractions

The high-pressure extractions were carried out using the apparatus and conditions described in our previous work on high-pressure extraction of cherry samples [12]. Briefly, supercritical CO_2_ was delivered to the extraction cell using a high-pressure liquid compressor (maximum pressure of 30 MPa) and EtOH was delivered by a high-pressure liquid pump (L-6200A, Hitachi, Merck Darmstadt, Germany). A stainless-steel extraction cell (~20 mL) was filled with dried apple residues (4.0 g) and a filter was placed on both endings of the cell to achieve a uniform distribution of the solvent flow, as well as to prevent line obstructions. Extraction cell was placed into a water bath with temperature controlled by an immersion circulator (±0.1 °C, DC30, Thermo Haake, Karlsruhe, Germany) and pressure was maintained by a back-pressure regulator (26-1762-24-090, Tescom, Selmsdorf, Germany) and measured by a pressure transducer (C204, Setra, Boxborough, MA, USA). Extracts were recovered in a recovering flask placed in an ice bath and the expanded CO_2_ flow was measured by a wet gas meter (DM3C ZE 1411, G.H: Zeal Ltd., London, UK). A two-step fractioned extraction methodology was employed, comprising (1) a first CO_2_ SFE step in order to remove the low polarity of the CO_2_-soluble compounds (15 min static + 60 min dynamic period) and (2) a second ESE extraction step, for 90 min, to extract more polar compounds, wherein mixtures of CO_2_ + ethanol (10–100%, *v*/*v*) were used as extraction solvent. All extractions were performed in the same apparatus at 50 °C and 25 MPa, which was close to the pressure limit of the equipment. Furthermore, the selection of extraction conditions, including extraction time, was based on previous studies with apple products [13,14], as well as with other fruit residues [11,12]. In particular, Massias et al. [14] successfully recovered phenolic compounds from apple peels using a CO_2_ + 25% mol cosolvent (ethanol at 96%) at 50 °C and 25 MPa, whereas Adil and co-authors [13] demonstrated that an extraction time at a value of 80 g of fluid/g of matrix was necessary to reach a plateau on the extraction curve for phenolic compounds from apple pomace. Additionally, it has been shown that a pre-treatment of raw material with supercritical CO_2_ is required to efficiently remove lipophilic and nonpolar substances. These process conditions improve the availability of phenolic compounds for the 2nd extraction step [11,12], where increasing concentrations of EtOH in the solvent mixture enhances the extraction of these compounds from fruit residues [11,12].

In this work, the outlet tubing line was cleaned with 50 mL of EtOH after the 2nd extraction step. The solid/solvent ratios for the 1st and 2nd steps were 1:47 ± 2 (*w*/*v*) and 1:67 ± 3 (*w*/*v*), respectively. The central point of the experimental design was made in triplicate to determine experimental error of the yield values. Ethanol-containing extracts were concentrated at 40 °C, under vacuum and in the dark, and kept at −20 °C until further analysis.

#### 2.3.2. Conventional Extractions

Conventional solid–liquid extractions were carried out with ethanol for comparison purposes. The solid/solvent ratio was 1:10 (*w*/*v*) and the extraction was carried out over 2 h at 50 °C. Afterwards, the extracts were concentrated at 40 °C under vacuum and in absence of light and kept at −20 °C until further analysis.

### 2.4. Phenolic Profile and Total Flavonoid Contents—HPLC Analysis

HPLC analysis of phenolic compounds was carried out using a Surveyor equipment from Thermo Finnigan with a diode array detector (DAD) (Thermo Finnigan—Surveyor, San Jose, CA, USA), as described in our previous work [3,5]. Briefly, separations were performed at 35 °C with a LiChrospher C18 column (5 µm, 250 mm × 4 mm i.d.; Merck AG, Germany) with a guard cartridge of the same type. A mobile phase constituted by phosphoric acid 0.1% (*v*/*v*) (eluent A) and a mixture of phosphoric acid:acetonitrile:H_2_O 1:400:599 (*v*/*v*/*v*) (eluent B) was used with a discontinuous gradient of 0–20% B (0–15 min), 20% B (15–25 min), 20–70% B (25–70 min), 70% B (70–75 min), 70–100% B (75–85 min) and 100% B (85–90 min), at a flow rate of 0.7 mL/min. Diode array detection was performed between 200 and 800 nm and the data acquisition system was Chromquest version 4.0 (Thermo Finnigan–Surveyor, San Jose, CA, USA). Extracts were diluted in EtOH and microfiltered (0.22 µm) before HPLC injection. The identification of compounds was conducted by comparing retention time, spectra and spiking samples with pure standards. The total chromatographic area recorded at 360 nm was used to quantify the total flavonoid contents of the apple extracts and a calibration curve with quercetin was performed to express the results in milligrams of quercetin equivalents (QE) per gram of extract.

### 2.5. Folin–Ciocalteu Assay

The total concentration of phenolic compounds present in the apple extracts was determined according to the modified Folin–Ciocalteu colorimetric method, as described in a previous study [15]. Results were expressed in mg of gallic acid equivalents (GAE) per g of extract.

### 2.6. Oxygen Radical Absorbance Capacity (ORAC) Assay

The ORAC assay was carried out following the method of Huang et al. [16], modified for the FL800 microplate fluorescence reader (Bio-Tek Instrument, USA), as described in a previous work [3]. This assay measures the ability of samples to inhibit the oxidation of disodium fluorescein catalyzed by peroxyl radicals generated from AAPH, using Trolox for the calibration curve. The results are presented as micromoles of Trolox equivalents antioxidant capacity (TEAC) per gram dry weight (dw) and are a mean ± SD of six replicates.

### 2.7. Hydroxyl Radical Adverting Capacity (HORAC) Assay

The HORAC assay was based on the method in Ou et al. [17], modified for the FL800 microplate fluorescence reader, as described previously in our work [18]. This assay evaluates the hydroxyl radical prevention capacity of a sample using (i) a Fenton-like reaction with CoF_2_ and H_2_O_2_ to generate the radicals, (ii) fluorescein as a probe and (iii) caffeic acid as the standard for the calibration curve. The results are expressed as micromoles of caffeic acid equivalents (CAEAC) per gram of dry weight (dw) and are a mean ± SD of six replicates.

### 2.8. Hydroxyl Radical Scavenging Capacity (HOSC) Assay

The HOSC assay was performed according to Moore and co-authors [19], using a FL800 microplate fluorescence reader and as described in a previous work [20]. This assay evaluates the hydroxyl radical scavenging capacity of a sample using fluorescein as a probe and a classic Fenton reaction with Fe(III) and H_2_O_2_ as a source of hydroxyl radicals. Trolox was used for the calibration curve and results are expressed as micromoles of Trolox equivalent antioxidant capacity (TEAC) per gram of extract (dw). Results are presented as a mean ± SD of six replicates.

### 2.9. Cell-Based Assays

#### 2.9.1. Cell Culture

Human colon cancer cell line, Caco-2, were obtained from Deutsche Sammlung von Microorganismen und Zellkulturen (DSMZ, Germany). This cell line was cultured in an RPMI 1640 medium supplemented with 10% of FBS. Stock cells were maintained as monolayers in 175 cm^2^ culture flasks and incubated at 37 °C with 5% CO_2_ in a humidified atmosphere.

Undifferentiated NTera-2/clone D1 (NT2) cells, obtained from the American Type Culture Collection (ATCC), were routinely propagated in 2D culture systems [21] and the 3D neural differentiation was performed in an agitation-based culture system as previously described [22,23]. Briefly, undifferentiated NT2 cells were inoculated as a single cell suspension in 125 mL spinner vessels equipped with a ball impeller (Wheaton) in DMEM supplemented with 10% (*v*/*v*) FBS and 1% (*v*/*v*) P/S. After 3 days of cell aggregation, neuronal and astrocytic differentiation was induced by addition of 10 µM RA, with a 50% media exchange every 2–3 days for 21 days. Following this period, a 3D neuron–astrocyte co-culture (neurospheroids) was obtained and maintained in DMEM supplemented with 5% (*v*/*v*) FBS and 1% (*v*/*v*) P/S up to day 50; these neurospheroids were harvested between days 38 and 50 of culture and used for neurotoxicity and antioxidant activity assays.

#### 2.9.2. Cytotoxicity Evaluation in Confluent Caco-2 Cells

Cytotoxicity assays were performed as described by previously [12] with some modifications. Assays were carried out using confluent and non-differentiated Caco-2 cells, as this cell model shares some characteristics with crypt enterocytes, being a well-established intestinal model to evaluate the effect of natural and synthetic compounds on intestinal function [24,25]. Briefly, cells were seeded at a density of 2 × 10^4^ cell/well in 96-well culture plates and allowed to grow for 7 days, with medium renewal every 48 h. After that, Caco-2 cells were incubated with apple extracts, prepared in EtOH, diluted in PBS (concentration range of 5–50 mg/mL). Control wells were prepared by incubating the cells with PBS and PBS + EtOH (1%). After 1 h of incubation, the medium was removed and 100 µL of the colorimetric reagent MTT (0.5 mg/mL) diluted in RPMI 1640 medium supplemented with 0.5% of FBS was added to each well and left for 4 h. The reaction was stopped with DMSO (150 µL/well) and formazan formation was quantified by measuring the absorbance at 570 nm with a SPECTRAmax TM microplate reader (Molecular Devices Corporation, Sunnyvale, USA). The percentage of cell viability was calculated using the absorbance values relative to the control wells. For each extract, three independent experiments were performed in triplicate.

#### 2.9.3. Cellular Antioxidant Activity (CAA) in Confluent Caco-2 Cells

Cellular antioxidant activity was measured according to the methodology optimized by Wolfe and Liu [26] and adapted to a Caco-2 cell line as described in our previous work [27,28]. Briefly, cells were seeded at a density of 2 × 10^4^ cell/well on a 96-well plate and assays were performed with confluent monolayers obtained after 7 days of culture (medium exchange was performed every 48 h). After that, the medium was removed and the cells were washed twice with PBS. Triplicate wells were treated for 1 h with 100 μL of different concentrations of apple extracts (5–50 mg/mL) or quercetin (1.25–20 µM) plus 25 μM DCFH-DA diluted in PBS. Then, the medium was removed and replaced by PBS containing 600 μM AAPH. The 96-well microplate was placed into a fluorescence reader (FL800, Bio-Tek Instruments, USA) at 37 °C. Emission at 530 ± 25 nm was measured after excitation at 485 ± 20 nm every 5 min for 1 h. Each plate included triplicate control wells (cells treated with DCFH-DA and an oxidant, namely, AAPH) and blank wells (cells treated with DCFH-DA without an oxidant). Quercetin was used as a standard. The CAA of extracts was quantified according to Wolfe and co-authors [26]. The EC_50_ values are stated as mean ± SD for triplicate sets of data obtained from the same experiment. EC_50_ values were converted to CAA values expressed as micromoles of quercetin equivalents per gram of apple extract. Results are presented as a mean of three independent experiments.

#### 2.9.4. Cytotoxicity Evaluation in 3D Neuron–Astrocyte Aggregates

Cytotoxicity evaluation of apple extracts in 3D neuron–astrocyte neurospheroids was performed as described previously [21]. Briefly, neurospheroids were harvested from spinner vessels between days 38 and 50 of culture, where aggregate diameter was kept stable, typically at approximately 180 µm, and were seeded in 96-well plates at 10 aggregate/well. Cells were incubated in DMEM supplemented with 5% (*v*/*v*) FBS and 1% (*v*/*v*) P/S, before carrying out neurotoxicity evaluation. Six wells were used per test condition and the culture medium was used as an untreated control. Cell viability was initially evaluated by the Presto blue™ cell viability assay (Life Technologies) according to the manufacturer’s instructions. Briefly, Presto blue cell viability reagent was diluted 1:10 in culture media and incubated with aggregates for 40 min at 37 °C and 5% CO_2_. The fluorescence intensity was evaluated using a FluoroMax^®^ -4 spectrofluorometer, with excitation and emission wavelengths of 590/20 nm and 560/20 nm, respectively. Neurotoxicity of the selected apple extract and quercetin was further evaluated for concentrations of 31.25–500 mg/L and 2.5–40 mg/L, respectively. The different concentrations of apple extract and quercetin were added to the neurospheroids for 24 h. After that, the medium was removed and cell viability of each well was further evaluated by the Presto blue™ cell viability assay as described above. The percentage of cell viability was calculated using the following equation:

(1) $$\%\;cell\;viability=\frac{\left(F_{S}\left(t_{f}\right)-F_{B}\left(t_{f}\right)\right)/\left(F_{S}\left(t_{0}\right)-F_{B}\left(t_{0}\right)\right)}{\left(F_{C}\left(t_{f}\right)-F_{B}\left(t_{f}\right)\right)/\left(F_{B}\left(t_{0}\right)-F_{B}\left(t_{0}\right)\right)}\times 100\%$$

where *F*_S_, *F*_B_ and *F*_C_ are the fluorescence intensity of test sample, blank (Presto blue™ diluted in culture media without cells) and control (cells cultured in culture medium), respectively, recorded at the beginning (*t*_0_) and at the end (*t*_f_) of the incubation time with samples.

#### 2.9.5. Inhibition of ROS Generation in 3D Neuron–Astrocyte Neurospheroids

These assays were performed according to the method described by Wolfe and co-authors [26] which was modified for the 3D neuron–astrocyte neurospheroids [22]. Briefly, neurospheroids were harvested from spinner vessels between days 38 and 50 of culture, where aggregate diameter was kept stable, typically at approximately 180 µm, and were distributed in 96-well plates at 10 aggregate/well. After 24 h, the medium was removed, the cells were washed with pre-warmed PBS (10 mM, pH of 7.4). Then, the cells were incubated with samples, namely apple extract (31.25 and 62.5 mg/L) or quercetin (2.5 and 5 mg/L), together with the probe (40 µM DCFH-DA) diluted in cell culture medium—DMEM-HG without phenol red (Invitrogen^®^), supplemented with 5% FBS, 1% PenStrep, 1 mM sodium pyruvate and 4 mM L-glutamine. After 1 h of incubation, the medium was removed and cells were incubated with the stress inducer, namely, 246 µM tert-butyl hydroperoxide (t-BHP). The 96-well microplate was placed into a fluorescence reader (FL800, Bio-Tek Instruments, USA) at 37 °C. Emission at 530 ± 25 nm was measured after excitation at 485 ± 20 nm every 5 min for 1 h. Each plate included six replicate control wells (cells treated with DCFH-DA only and t-BHP) and blank wells (cells treated with DCFH-DA without t-BHP). The antioxidant activity of the samples was calculated using the area under the curve as described previously [26] and the capacity of samples to decrease the intracellular ROS was expressed in terms of percentage relative to the control. Results were presented as mean ± SD of five independent experiments. Statistical analysis of the results was carried out using GraphPad Prism 6.03 (GraphPad Software, Inc., San Diego, USA). A one-way ANOVA analysis with Tukey’s post-hoc multiple comparison test was performed to assess statistical differences between samples and controls.

## 3. Results and Discussion

The extraction of phenolic compounds from apple residues using high-pressure methodologies was only reported in two studies. In the first study, Adil and co-authors optimized the extraction conditions to improve the recovery of total phenolics from apple pomace skin and pulp residues of Starking and Amasya apple varieties that remain after pressing the fruits for juice production) by testing a range of CO_2_ + ethanol concentrations (14–20%), extraction times (10–40 min), pressures (20–60 MPa) and temperatures (40–60 °C) [13]. Later on, Massias et al. identified and quantified the major phenolics extracted from Golden apple peels and monitored the extraction kinetics of these compounds using CO_2_:ethanol 73:27 *v*/*v*, at 25 MPa and 50 °C. Taking into account this previous knowledge, in our study, we further evaluated the potential of high-pressure technology to extract phenolic compounds from apple residues and explored the impact of higher compositions of ethanol (up to 100%) after a pre-treatment of raw material with supercritical CO_2_, on the development of antioxidant rich fractions. The experiments were carried out with the residues of BE apple (ripe fruits that were not suitable to sell due to appearance defects), which is a traditional Portuguese variety that presents high content of phenolic compounds, including catechin, epicatechin and quercetin glucosides [3].

### 3.1. Impact of Extraction Conditions on Yield and Phenolic Content of BE Residues Extracts

**Figure 1:**
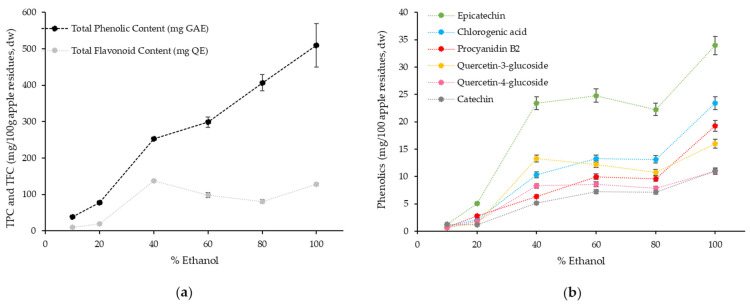
Effect of ethanol (EtOH) concentration on phenolic compounds extracted from BE apple residues with high-pressure technology: (**a**) total phenolics and total flavonoids recovery (mg/100 g raw material, dw); (**b**) individual phenolics recovery (mg/100 g raw material, dw).

Table 1 shows the extraction yield, phenolic content and composition, as well as the antioxidant activity of all apple extracts obtained using the two-step high-pressure extraction methodology and the conventional solid–liquid extractions performed in parallel with pure ethanol. Figure 1 presents the impact of ethanol concentration on the recovery of phenolic compounds from Bravo de Esmolfe apple residues. The total phenolic content was estimated using the Folin–Ciocalteu assay to enable a direct comparison with data from the literature [13]. However, since this method has some interferences with other compounds (e.g., amino acids, peptides, reduction sugars and ascorbic acid) [29], a HPLC-DAD method was also used to quantitate individual phenolics and total flavonoids present in the samples.

Our results show that a lower extraction yield (0.9 ± 0.4%) was achieved in the first step (Table 1), where nonpolar and lipophilic substances were preferentially extracted; the extract obtained (Extract A in Table 1) contained the lowest content of total phenolic (TPC) and flavonoid (TFC).

At the pressure and temperature conditions used in the second step (25 ± 0.4 MPa, 50 ± 0.1 °C) and for the solvent compositions tested, single homogeneous supercritical phases were always attained, according to what was described previously by Durling et al. [30]. As expected, the extraction yields (Table 1) and the recovery of phenolic compounds from BE apple residues (Figure 1) were positively affected by the concentration of ethanol in the solvent mixture. This result might be associated with the increase in covalent (hydrogen bonding) and dipole–dipole interactions that enhance the solubility of apple phenolic compounds, as described before by several authors [13,31,32], rather than the solvent density, which slightly decreased with the increase in ethanol percentage in the solvent mixture [33]. The extraction with high-pressure pure ethanol (Extract G) generated the highest extraction yield (80.8% *w*/*w*; Table 1) and promoted the highest recovery of phenolic compounds (509 ± 8 mg GAE/100 g of Bravo de Esmolfe residues, Figure 1a). The recovery of flavonoids was maximized for the extraction conditions performed from 40% of ethanol onwards (80.8–137.4 mg QE/100 g Bravo de Esmolfe residues; Figure 1a).

The identification of specific phenolic compounds in apple extracts was performed by HPLC-DAD and results confirm that the increase in ethanol concentration generally improved the extraction of epicatechin, catechin, chlorogenic acid, procyanidin B2 and quercetin derivatives (Figure 1b). This result is in agreement with other studies showing that the solubility of phenolic compounds, namely, quercetin, catechin and epicatechin, increases with the increase in ethanol concentration [31,32,34]. Accordingly, Adil and colleagues obtained higher recovery of phenolic compounds from apple pomace (obtained from mixture of Starking and Amasya varieties) when using the highest percentage of ethanol (20%) in the subcritical extraction process [13]. In our work, the highest recovery of the individual phenolic compounds was found for high-pressure pure ethanol and the amounts extracted (Figure 1b) were similar, or slightly lower, than the values reported by Massias et al. when applying a subcritical fluid extraction process (25 MPa, 50 °C, CO_2_ + ethanol (75:25 mol ratio)) to dried apple peels from Golden variety—6–36 mg/100 g dry peel for catechin and chlorogenic acid and 55–140 mg/100 g dry peel for epicatechin and quercetin derivatives [14]. These authors also showed similar extractions yields for phloridzin (6–36 mg/100 g dry peel), which is another representative phenolic compound from apples recognized as a quality marker of apple pomace by-products [35] and a compound with known health benefits, such as anti-hyperglycemic potential [36]. In our work, the content of phloridzin was not quantified in the apple extracts as BE contains very low concentrations of this phenolic compound (<0.6 mg/100 g fw apple) when compared with other apple cultivars [5].

It is important to note that the extraction yield and the recovery of phenolic compounds from BE residues was lower when using a conventional extraction with ethanol as solvent (yield, 23.9%; total phenolic recovery, 90.8 mg GAE/100 g dw; total flavonoid recovery, 27.9 mg QE/100 g dw; Extract H; Table 1), indicating that the use of high-pressure technology improved the recovery of phenolic compounds from these agro-food residues. This could be explained by several factors, such as (i) the slightly increase of the ethanol density in the P, T conditions applied (from 0.7857 to 0.7863 kg/L) and (ii) the use of a first step with supercritical CO_2_ (15 min static + 60 min dynamic period), which is described to be efficient in removing lipophilic and nonpolar substances, making high polarity phenolic compounds more available for extraction on the 2nd step [10,12].

Among all apple extracts, samples B and D presented the highest phenolic content (>7 mg GAE/g dw; Table 1) which could be explained by the selectivity of the solvent mixture, since higher percentages of ethanol can also promote the extraction of other compounds besides phenolics.

The differences observed between the phenolic recovery obtained in our study with the ones reported by Asil et al. [13] and Massias et al. [14] could be related not only to the different extraction conditions applied but also to the distinct phenolic content of the raw materials [3] and type of by-products [35,37]. In our study, we used the ripe fruits that were not commercially available from an apple variety rich in phenolic compounds [3]. In the future, the use of unripe apples (fruits discarded in the orchards by thinning or natural drop) can also be considered for the development of phenolic-rich extracts, as they contain higher phenolic content than ripe fruits [36,38]. Additionally, other drying methods can be explored to improve the economic viability of the process. In fact, we used the freeze-drying method to ensure the phytochemical quality of the raw material [39], but other processes, such as convectional hot-air drying or microwave drying should be studied to reduce the costs in industrial applications [40].

### 3.2. Antioxidant Activity of BE Residues Extracts

The antioxidant activity of BE residues extracts was determined using three different and complementary chemical assays. The ORAC and HOSC assays measure the ability of samples to scavenge peroxyl and hydroxyl radicals [16,19], whereas HORAC evaluates the capacity of samples to prevent the generation of hydroxyl radicals [17]. As shown in Table 1, the extracts C, D and E (obtained with ethanol percentages from 20% to 60%) presented the highest values of HORAC, ORAC and HOSC, respectively; this effect could be related to the phenolic composition of each sample. In fact, high correlations were obtained between epicatechin and procyanidin B2 with ORAC and HORAC values, respectively (Table 2), indicating that these compounds could be the main contributors of the antioxidant capacity of BE extracts. Epicatechin is the phenolic compound present in the highest quantity in the BE extracts (Table 1) and was already identified to display high ORAC values among other flavonoids [41]. Procyanidin B2 was also pointed to be, together with epicatechin, the most important antioxidant presents in both apple peel and apple flesh, showing high correlation with other antioxidant methods, such as ferric reducing/antioxidant power (FRAP) and the β-carotene−linoleic acid model system (β-CLAMS) [42]. In contrast, extracts A and H presented the lowest antioxidant activity due to the lower content of phenolic compounds (Table 1). This result reinforces the use of high-pressure technology with CO_2_ and ethanol mixtures to develop antioxidant-rich extracts from apple residues.

The antioxidant activity of all apple extracts developed by high-pressure technology was also evaluated at a cellular level to better predict their bioactive potential as some of the processes related with uptake, distribution and metabolism of antioxidant compounds are addressed [26]. Assays were performed in confluent Caco-2 cells, a well-established human cell model of the intestinal barrier to evaluate the cellular the antioxidant capacity of phenolics from foods [43]. Cytotoxicity of samples was also analyzed and none of extracts showed cytotoxic effect in Caco-2 cells. The highest CAA value was obtained for extract D (1.50 µmol QE/g dw) (Table 1) probably due to the high content of flavonoids (Table 1) that present higher lipophilicity than phenolic acids, which leads to higher cell-membrane permeability [44]. Among all flavonoids, the highest correlation was obtained for epicatechin (R = 0.710; Table 2) reinforcing the contribution of this flavonoid to the antioxidant capacity of apple extracts at a cellular level. In fact, epicatechin was already reported to reduce ROS levels in Caco-2 cells [45] and to modulate the expression of genes involved in the cellular response to oxidative stress (STAT1, MAPKK1, MRP1 and FTH1) [46]. It is important to mention that, when estimating CAA values in terms of total phenolics, the results obtained ranged between 2.22 and 3.98 µmol QE/100 µmol of total phenolics, which are similar to the CAA values reported by Wolfe and co-authors in the HepG2 cell line (1.45–3.07 µmol QE/100 µmol of total phenolics) [26]. Our data also show good correlations (r > 0.65) between CAA and both ORAC and HORAC assays (r > 6.5), indicating that apple extracts that exhibit high scavenging capacity of peroxyl radicals and/or high inhibition of hydroxyl radicals can promote a high cellular antioxidant response.

### 3.3. Antioxidant Activity in 3D Neuron–Astrocyte Neurospheroids

**Figure 2:**
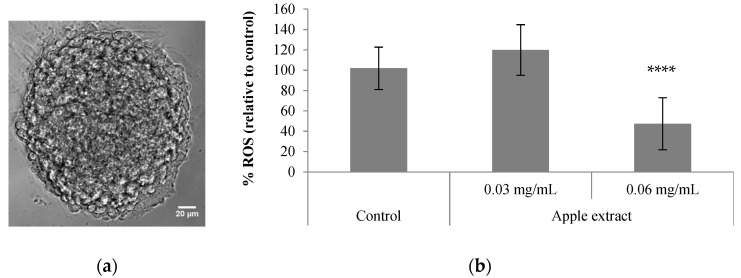
In vitro neuroprotective effect of Bravo de Esmolfe apple extract—a 3D cell model approach: (**a**) phase contrast image of human differentiated neurospheroids derived from NT2 cells; (**b**) effect of apple extract on ROS levels present in neurospheroids incubated with t-BHP (256 µM). Results shown are means of at least 5 replicates ± SD; *p* < 0.05 was considered as statistically significant (**** *p* < 0.0001 is relative to the control).

We also evaluated the neuroprotective potential of BE extract D, which was the one that showed the highest cellular antioxidant activity (Table 1), in reducing the ROS levels in 3D aggregates of differentiated human NT2 cells (so-called neurospheroids; Figure 2a). These neurospheroids are mainly composed of glial cells, namely functional astrocytes (approximately 80% of total cells) and neurons and are thus a robust cell model to evaluate human neuronal and astrocytic toxicity [23]. In previous studies, these neurospheroids were successfully applied to screen the neuroprotective effect of chemically synthesized compounds [22], as well as blackberry-digested phenolic compounds [47].

Our results demonstrated that the BE extract D, at a concentration of 0.06 mg/mL, significantly reduced ROS formation in neurospheroids submitted to an oxidative stress induced by t-BHP (Figure 2b). These results were compared with quercetin alone, a phenolic that presents neuroprotective effect in various models of neuronal injury and neurodegenerative diseases [48,49]. Despite its low bioavailability, this compound is able to permeate the blood–brain barrier and the main mechanisms associated with neuroprotection are due to its direct antioxidant capacity and the modulation of signaling pathways that stimulates cellular defenses against oxidative stress (e.g., Nrf2-ARE, PON2, JNK and TNF-α) [48,49]. In our study, the percentage of ROS reduction by the BE extract D is within the range of values obtained for the quercetin alone tested at concentrations of 2.5 mg/L (60.9%) and 5 mg/L (39.5%), suggesting that the main bioactive compounds of the extract present promising neuroprotective effect. Noteworthy, all the concentrations of BE extracts and quercetin tested were not cytotoxic in this 3D cell model, as confirmed using cell viability assays (Figure S1). Future studies should include the evaluation of the impact of other phenolic compounds from BE extract on ROS reduction and modulation of cell signaling pathways related with neuroprotection to unveil the protective effect of these apple phenolics against neurodegenerative diseases. Despite no cytotoxic effects of the apple extract were observed in neither human cell line, future work aiming at the industrial development of these extracts for pharma or nutraceuticals applications should consider the removal of the seeds from the apple residues to eliminate the presence of amygdalin (cyanogenic glycoside), which is potentially toxic [35,36].

Our results are in line with previous studies supporting the idea that apple fruits and apple-related products display a protective effect against neurodegenerative diseases. Tchanchou and co-authors showed that the administration of apple juice concentrate (0.5% diluted in drinking water) for 1 month, induced significant improvements in cognitive-related performance and reduced the pro-oxidative status in a mouse model of neurodegeneration [50]. Additional work from this group using mice with genetically induced oxidative stress (an ApoE-deficient strain) confirmed that 1 month of apple juice concentrate intake reduced the accumulation of ROS in brain tissue and attenuated cognitive impairment [51,52]. More recently, the antioxidant and anti-inflammatory-mediated neuroprotective properties of apple ciders and juice was demonstrated using cell-based assays (monolayers of SH-SY5Y and BV2 cells) and in vivo models [53]. In particular, Alvariño et al. (2020) demonstrated that the mice group treated with these beverages presented reduced brain oxidative stress and inflammatory markers after LPS injection. Genetic expression of antioxidant enzymes and glutathione levels were also greatly augmented after drink intake, supporting the protective role of apple compounds in neurodegenerative disorders [39].

## 4. Conclusions

In this work, we applied high-pressure technology to generate antioxidant-rich fractions from residues of BE apple variety, through modulation of key process parameters. By performing a first extraction step with supercritical CO_2_, followed by ESE with different mixtures of CO_2_ and ethanol (10–100%, *v*/*v*), we efficiently generated apple extracts with different phenolic compositions and antioxidant activities. The apple extract obtained with CO_2_ + ethanol (40%, *v*/*v*) exhibited the highest content of flavonoids, as well as the highest antioxidant activity potential, confirming that epicatechin and procyanidin B2 were the major contributors of the bioactive effect. This extract obtained from BE residues also showed neuroprotective effects in a human 3D cell model-based study through inhibition of the production of free radicals in neuron-astrocyte neurospheroids derived from NT2 cells. Although further studies are needed to unveil the mechanisms of action of apple extracts in neuroprotection effects, this work is a step forward in the development of antioxidant-rich fractions from residues of BE apple, adding potential high value to these agro-food by-products.

## Figures and Tables

**Table 1:** Bravo de Esmolfe extracts obtained using high-pressure technology and conventional solid–liquid ethanol extraction; experimental conditions, global yields, phenolic contents and antioxidant activities were estimated for each extract.

Extract ID	Solvent Mixture CO_2_: EtOH	Yield (%)	Phytochemical Characterization (mg/g dw)								Antioxidant Activity (µmol/g dw)			
			TPC ^1^	TFC ^2^	Cat ^3^	CAc ^4^	Ep ^5^	Q3g ^6^	Q4g ^7^	PB2 ^8^	ORAC ^9^	HOSC ^10^	HORAC ^11^	CAA ^12^
Fractioned High-Pressure Extraction (25 ± 0.4 MPa, 50 ± 0.1 °C)														
1st Step: Supercritical CO_2_ extraction (extraction time, 60 min; solid/solvent ratio, 1:47 ± 2)														
A	100:0	0.9 ± 0.4	4.9 ± 0.7	0.98 ± 0.05	0.11 ± 0.01	0.16 ± 0.01	0.21 ± 0.01	0.15 ± 0.01	0.14 ± 0.01	0.16 ± 0.01	65 ± 8	64.9 ± 6.8	<2.5	<0.08
2nd Step: Enhanced solvent extraction (pressure, 25 ± 0.4 MPa, temperature, 50 ± 0.1 °C; time, 90 min; solid/solvent ratio, 1:67 ± 3)														
B	90:10	4.4 ± 0.4	8.8 ± 1.1	2.17 ± 0.11	0.28 ± 0.01	0.21 ± 0.01	0.29 ± 0.01	0.17 ± 0.01	0.15 ± 0.01	0.16 ± 0.01	200 ± 26	86 ± 8	14 ± 1	1.16 ± 0.22
C	80:20	12.7	6.1 ± 0.3	1.54 ± 0.08	0.09 ± 0.01	0.14 ± 0.01	0.40 ± 0.02	0.11 ± 0.01	0.14 ± 0.01	0.22 ± 0.01	197 ± 19	145 ± 23	102 ± 3	1.43 ± 0.38
D	60:40	36.1	7.0 ± 0.4	3.81 ± 0.19	0.14 ± 0.01	0.28 ± 0.01	0.65 ± 0.03	0.37 ± 0.02	0.23 ± 0.01	0.18 ± 0.01	285 ± 16	134 ± 10	63 ± 9	1.50 ± 0.24
E	40:60	56.3	5.3 ± 0.4	1.74 ± 0.09	0.13 ± 0.01	0.23 ± 0.01	0.44 ± 0.02	0.22 ± 0.01	0.15 ± 0.01	0.18 ± 0.01	244 ± 10	202 ± 27	60 ± 5	1.07 ± 0.04
F	20:80	66.6	6.1 ± 0.9	1.22 ± 0.06	0.11 ± 0.01	0.20 ± 0.01	0.33 ± 0.02	0.16 ± 0.01	0.12 ± 0.01	0.14 ± 0.01	147 ± 22	120 ± 18	33 ± 2	1.23 ± 0.03
G	0:100	80.8	6.3 ± 0.1	1.58 ± 0.08	0.14 ± 0.01	0.29 ± 0.01	0.42 ± 0.02	0.20 ± 0.01	0.14 ± 0.01	0.24 ± 0.01	123 ± 14	118 ± 15	94 ± 2	1.19 ± 0.23
Conventional Extraction (50 ± 0.1 °C; extraction time 120 min)														
H	0:100	23.9	3.8 ± 0.1	1.17 ± 0.06	0.02 ± 0.01	0.06 ± 0.01	0.14 ± 0.01	0.11 ± 0.01	0.10 ± 0.01	0.13 ± 0.01	117 ± 6	27 ± 3	18 ± 2	nd

^1^ Total phenolic content determined with the Folin–Ciocalteu method (results are expressed as mg of gallic acid equivalents per g of dried extract). ^2^ Total flavonoid content determined by the total chromatographic area recorded at 360 nm (results are expressed mg of quercetin equivalents per g of extract). ^3^ Catechin content. ^4^ Chlorogenic acid content. ^5^ Epicatechin content. ^6^ Quercetin-3′-Glucoside content. ^7^ Quercetin-4′-Glucoside content. ^8^ Procyanidin B2. ^9^ Oxygen radical absorbance capacity value (µmol TEAC/g dw). ^10^ Hydroxyl radical scavenging capacity (µmol TEAC/g dw). ^11^ Hydroxyl radical adverting capacity (µmol CAEAC/g dw). ^12^ Cellular antioxidant activity (µmol QE/g dw). nd, not determined.

**Table 2:** Correlation coefficients between phenolic content and antioxidant activity of BE apple residues extracts.

	ORAC	HOSC	HORAC	CAA
TPC	0.392	−0.257	−0.106	0.499
TFC	0.801	0.190	0.179	0.553
Cat	0.239	−0.298	−0.388	0.141
CAc	0.426	0.309	0.398	0.467
Ep	0.799	0.577	0.596	0.710
PB2	0.031	0.275	0.872	0.375
Q3g	0.629	0.223	0.075	0.323
Q4g	0.690	0.158	0.111	0.295
CAA	0.731	0.532	0.657	-

TPC, total phenolic content; TFC, total flavonoid content; Cat, Catechin; CAc, Chlorogenic acid; Ep, Epicatechin; PB”, Procyanidin B2; Q3g, Quercetin-3-glucoside; Q4g, Quercetin-4-glucoside; ORAC, oxygen radical absorbance capacity value; HOSC, hydroxyl radical scavenging capacity (µmol TEAC/g dw); HORAC, hydroxyl radical adverting capacity (µmol CAEAC/g dw); CAA, cellular antioxidant activity.

## Data Availability

The data supporting the findings of this study are available within the article and its supplementary material.

## Supplementary Materials

The following are available online at https://www.mdpi.com/article/10.3390/antiox10091469/s1, Figure S1: Effect of different concentrations of quercetin (a) and BE extracts (b) on cell viability of human differentiated neurospheroids derived from NT2 cells.
