# Supplementary material for: Using High-Pressure Technology to Develop Antioxidant-Rich Extracts from Bravo de Esmolfe Apple Residues
Source: Antioxidants (Basel). 2021 Sep 15;10(9):1469. doi: 10.3390/antiox10091469 (PMC8468280; doi:10.3390/antiox10091469)
Supplement: Supplementary file 1 [file antioxidants-10-01469-s001.zip › antioxidants-1334174-supplementary.pdf]

Supplementary Material

Using High-Pressure Technology to Develop Antioxidant-Rich Extracts from Bravo de Esmolfe Apple Residues

Mário Bordalo, Inês J. Seabra, Andreia Bento Silva, Ana Paula Terrasso, Catarina Brito, Margarida Serra, Maria R. Bronze, Catarina M. M. Duarte, Mara E. M. Braga, Hermínio C. de Sousa and Ana Teresa Serra

1. Supplementary Figures

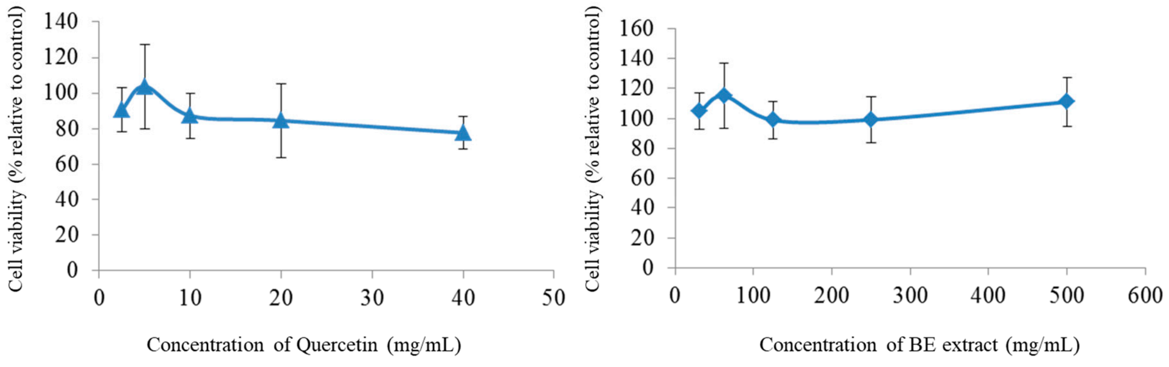

(**a**) (**b**)

Figure S1. Effect of different concentrations of quercetin (a) and BE extracts (b) on cell viability of human differentiated neurospheroids derived from NT2 cells.
